# Prime Editing Mediated Generation and Correction of the mdx5cv Mutation Restores Dystrophin Expression in Myoblasts

**DOI:** 10.3390/ijms27156927

**Published:** 2026-08-01

**Authors:** Ayesha Siddika, Fatima El Husseiny, Joël Rousseau, Jacques P. Tremblay

**Affiliations:** 1 Department of Molecular Medicine, Université Laval, Quebec, QC G1V 0A6, Canada; ayesha.siddika@crchudequebec.ulaval.ca (A.S.); fatima.el-husseiny.1@ulaval.ca (F.E.H.); 2 CHU de Québec Research Center, Université Laval, Quebec, QC G1V 4G2, Canada; joel.rousseau@crchul.ulaval.ca

**Keywords:** Duchenne muscular dystrophy, *Dmd* gene, prime editing, mdx5cv, dystrophin, point mutation, myoblasts, genome editing

## Abstract

Duchenne muscular dystrophy (DMD) is caused by mutations in the *DMD* gene that abolish dystrophin expression. Prime editing enables precise genome modification without generating double-strand DNA breaks or requiring donor DNA templates. We established an in vitro prime editing workflow to generate and subsequently correct the mdx5cv mutation in mouse C2C12 myoblasts. Following optimization of engineered prime editing guide RNAs (epegRNAs) and PAM-flexible prime editors, wild-type cells were edited, clonally isolated, and genotyped. Mutation correction was then evaluated using optimized epegRNA designs. Two rounds of prime editing introduced the mdx5cv mutation into approximately 20% of alleles in C2C12 cells creating the mdx5cv C2C12 cell line. Clonal isolation yielded five homozygous mutant clones among 59 expanded colonies. Optimization studies identified an epegRNA containing a 16 nucleotide reverse transcription template and a 10 nucleotide primer binding site (RTT16/PBS10) as the most efficient design. Correction of the pathogenic allele reached approximately 26%, whereas longer PBS lengths reduced editing efficiency. In silico off-target analysis using Cas-OFFinder identified no candidate genomic loci with fewer than three mismatches for the spacer sequences used in either mutation generation or correction, suggesting a favorable predicted specificity profile. Corrected mdx5cv C2C12 myoblasts retained their capacity to differentiate into multinucleated myotubes. Representative Western blot analysis detected dystrophin protein expression in differentiated corrected mdx5cv myotubes, consistent with successful correction of the pathogenic mutation. These findings establish a robust prime editing platform for both the generation and correction of the mdx5cv mutation and provide proof of concept that precise correction of the pathogenic mutation is associated with restoration of dystrophin expression following myogenic differentiation.

## 1. Introduction

Duchenne muscular dystrophy (DMD) is one of the most severe and common inherited neuromuscular disorders, affecting approximately 1 in 3500–5000 male births worldwide [1,2]. The disease is caused by mutations in the dystrophin gene (DMD; OMIM: 300377), located on chromosome Xp21.2, which encodes dystrophin, a critical cytoskeletal protein required for maintaining sarcolemmal stability during muscle contraction [3]. The human *DMD* reference transcript is RefSeq NM_004006.3, whereas the mouse *Dmd* transcript used in the present study corresponds to RefSeq NM_007868.6. Dystrophin forms a critical component of the dystrophin-associated glycoprotein complex, linking the intracellular actin cytoskeleton to the extracellular matrix and thereby protecting muscle fibers from contraction-induced damage [3,4]. Loss of functional dystrophin leads to progressive skeletal muscle degeneration, chronic inflammation, fibrosis, respiratory insufficiency, dilated cardiomyopathy, and premature mortality [5]. Despite significant advances in clinical management, including respiratory support and corticosteroid therapy, DMD remains a debilitating and ultimately fatal disorder with no curative treatment [5,6].

Current therapeutic strategies primarily aim to delay disease progression or partially restore dystrophin expression. Corticosteroids such as prednisone and deflazacort remain the standard of care and can temporarily improve muscle strength and prolong ambulation; however, long-term treatment is frequently associated with substantial adverse effects, including obesity, osteoporosis, growth retardation, and metabolic abnormalities [6,7]. More recently, mutation-specific therapies such as antisense oligonucleotide-mediated exon skipping and adeno-associated virus (AAV)-mediated micro-dystrophin gene replacement have demonstrated encouraging clinical outcomes [8,9,10,11,12]. Exon translation skipping agents restore the translational reading frame by excluding specific exons during pre-mRNA splicing, resulting in the production of shortened but partially functional dystrophin proteins [8,9]. Similarly, micro-dystrophin gene therapies have shown promising improvements in muscle function and dystrophin expression in clinical studies [10,11,12]. Nevertheless, these approaches remain limited by mutation specificity, repeated dosing requirements, immune responses, vector cargo constraints, and uncertainties regarding long-term efficacy and safety. Consequently, there is increasing interest in developing genome editing strategies capable of permanently correcting the endogenous *Dmd* gene.

The advent of CRISPR/Cas genome editing technologies has revolutionized the field of genetic medicine and opened new therapeutic possibilities for monogenic disorders such as DMD. Several studies have demonstrated that CRISPR/Cas9-mediated editing can restore dystrophin expression in cellular and animal models through exon deletion, exon skipping, exon reframing, and direct mutation correction strategies [13,14,15,16]. These approaches have achieved substantial dystrophin restoration and functional improvement in preclinical models, highlighting the therapeutic potential of genome editing for DMD. However, conventional CRISPR/Cas9 editing relies on the induction of double-strand DNA breaks (DSBs), which are repaired through endogenous DNA repair pathways such as non-homologous end joining (NHEJ) and homology-directed repair (HDR) [17]. Although effective, DSB mediated editing can result in unpredictable repair outcomes, including unwanted insertions and deletions (indels), large genomic deletions, chromosomal rearrangements, and activation of DNA damage responses [17,18]. Furthermore, HDR-dependent correction is generally inefficient in non-dividing cells, limiting its applicability in terminally differentiated skeletal and cardiac muscle tissues. These limitations have motivated the development of next-generation precision genome editing technologies that can introduce defined genetic modifications while minimizing unintended genomic alterations.

Prime editing has emerged as one of the most versatile and precise genome editing platforms developed to date. First described by Anzalone et al. in 2019, prime editing enables targeted nucleotide substitutions, insertions, and deletions without requiring donor DNA templates or generating double-strand DNA breaks [19]. The system consists of a Cas9 nickase fused to an engineered reverse transcriptase and an engineered prime editing guide RNA (epegRNA), which directs target recognition while simultaneously encoding the desired genetic modification. Following target recognition and nick formation, the reverse transcriptase copies the edited sequence defined by the RTT sequence, allowing precise modification of a broad range of genetic variants [19].

Since its introduction, substantial efforts have been devoted to improving prime editing efficiency and expanding its targeting range. Engineered pegRNAs (epegRNAs) containing structured RNA motifs have been developed to enhance guide RNA stability and improve editing outcomes [20]. Additional optimization strategies, including refinement of primer-binding site and reverse transcription template designs, manipulation of DNA repair pathways, and implementation of PE3 nicking strategies, have further increased editing efficiency in mammalian cells [20,21]. Moreover, PAM-flexible Cas9 variants have significantly expanded the number of targetable genomic loci by recognizing non-canonical PAM sequences that are not recognized by standard SpCas9 [22,23,24]. Collectively, these advances have broadened the applicability of prime editing and increased its therapeutic potential for the correction of disease-causing mutations associated with monogenic disorders.

Recent studies have demonstrated successful prime editing mediated correction of pathogenic dystrophin mutations in patient-derived cells and experimental DMD models, supporting the feasibility of this technology as a future precision medicine approach for Duchenne muscular dystrophy [25,26]. Nevertheless, editing efficiencies remain highly dependent on target sequence context, PAM accessibility, pegRNA architecture, and delivery strategy. Furthermore, robust and genetically defined in vitro disease models are essential for optimizing editing systems and evaluating functional rescue before translation to in vivo studies.

Among available DMD models, the mdx5cv mutation represents a clinically relevant point mutation within the *Dmd* gene that disrupts normal dystrophin expression and leads to a dystrophic phenotype [27]. Although mdx5cv mice have been widely used for DMD research, stable myogenic cell models carrying this mutation provide valuable experimental platforms for systematically evaluating editing efficiency, mutation correction, myogenic differentiation, and dystrophin restoration under controlled conditions.

In the present study, we established a comprehensive prime editing workflow for both the generation and correction of the mdx5cv mutation in mouse C2C12 myoblasts. We first engineered a homozygous mdx5cv disease cell model through repeated prime editing and clonal isolation. We then optimized epegRNA architecture for mutation correction using PAM-flexible prime editor variants and evaluated restoration of dystrophin expression following myogenic differentiation. Together, these studies provide a robust cellular platform for the development and optimization of prime editing-based therapeutic strategies for Duchenne muscular dystrophy.

## 2. Results

### 2.1. Generation of a Homozygous mdx5cv Myoblast Cell Line by Prime Editing

To establish a genetically defined cellular model for subsequent mutation-correction studies, we first investigated whether prime editing could be used to introduce the pathogenic mdx5cv mutation into wild-type C2C12 myoblasts. The mdx5cv mutation consists of an A>T substitution in exon 10 of the mouse *Dmd* gene, generating a cryptic splice donor site that disrupts normal dystrophin expression.

**Figure 1:**
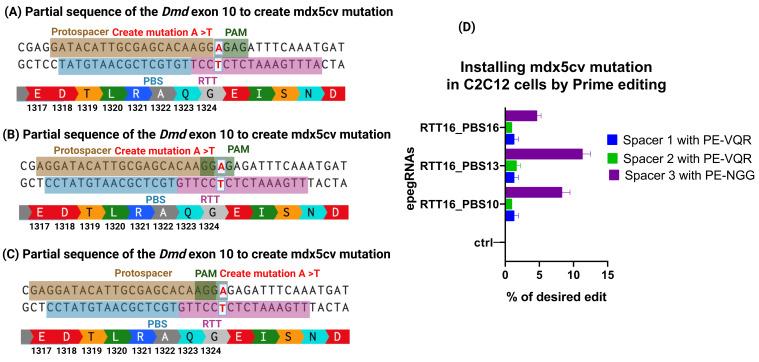
Prime editing mediated installation of the mdx5cv mutation in wild-type C2C12 myoblasts. (**A**–**C**) Schematic representation of the three editing strategies targeting exon 10 of the mouse *Dmd* gene. The pathogenic A>T substitution generating the mdx5cv allele is indicated in red. Protospacer sequences, PAMs, PBSs, and RTTs are shown. (**D**) Editing efficiencies of the three highest-performing epegRNAs selected from the 21 designs evaluated. Editing frequencies were determined 72 h after electroporation by Sanger sequencing and EditR analysis. Data are presented as mean ± SEM (N = 4 independent experiments).

A target region containing three nearby PAM sequences (NGAG, NGAG, and NGG) was selected to enable the evaluation of multiple prime editing configurations (Figure 1A–C). A total of 21 epegRNAs with varying reverse transcription template (RTT) and primer-binding site (PBS) lengths were designed and tested in combination with PAM-flexible prime editor variants (Table 1). All epegRNAs incorporated a 3′ tevopreQ1 stabilizing motif to enhance epegRNA stability and editing efficiency.

Prime editing efficiencies were assessed 72 h after electroporation by Sanger sequencing followed by EditR analysis. Editing activity varied substantially among the tested epegRNA designs, highlighting the strong influence of epegRNA architecture on editing outcomes at the *Dmd* exon 10 locus. For clarity, only the three highest-performing epegRNAs are shown in Figure 1D. The most efficient editing strategy achieved approximately 12% installation of the mdx5cv mutation in wild-type C2C12 myoblasts.

**Figure 2:**
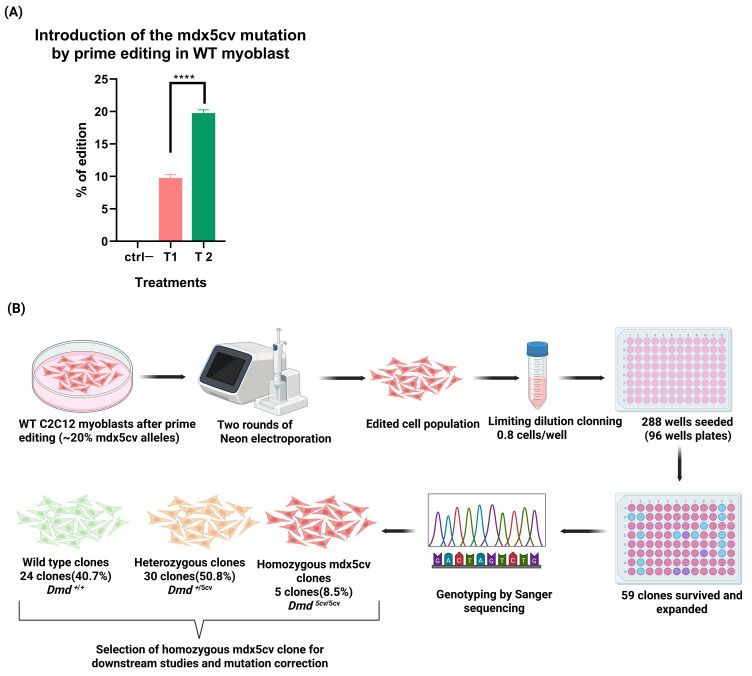
Generation of a homozygous mdx5cv C2C12 myoblast cell line by prime editing. (**A**) Two successive rounds of prime editing increased installation of the mdx5cv mutation to approximately 20% of alleles. **** *p* < 0.0001. (**B**) Workflow for clonal isolation and genotyping of edited cells. Limiting dilution cloning was performed at an average density of 0.8 cells per well across 288 wells. Among 59 expanded clones, 5 were homozygous for the mdx5cv mutation, 30 were heterozygous, and 24 remained wild-type. Image created with Biorender.com.

To generate a stable mutant cell line, wild-type C2C12 myoblasts were subjected to two successive rounds of prime editing using the optimized editing configuration (Figure 2A). Following the second treatment, the frequency of mdx5cv allele installation increased to approximately 20%, demonstrating cumulative enrichment of edited alleles.

The edited cell population was subsequently subjected to limiting dilution cloning at an average density of 0.8 cells per well across 288 wells. A total of 59 clones survived and expanded sufficiently for genotyping. Sanger sequencing identified 5 homozygous mdx5cv clones (8.5%), 30 heterozygous clones (50.8%), and 24 wild-type clones (40.7%) (Figure 2B). Representative sequencing chromatograms confirmed successful introduction of the pathogenic nucleotide substitution and establishment of homozygous mutant cell lines.

Collectively, these results demonstrate that prime editing can be used to efficiently generate a stable homozygous mdx5cv myoblast model through precise nucleotide substitution and clonal isolation. This engineered cell line provides a valuable platform for subsequent optimization of mutation-correction strategies and evaluation of dystrophin restoration.

### 2.2. Optimization of epegRNA Architecture at the Dmd Exon 10 Locus

To identify the most efficient epegRNA design for subsequent correction of the mdx5cv mutation, we evaluated prime editing mediated introduction of a silent PAM-disrupting mutation in wild-type C2C12 myoblasts. The same panel of nine epegRNAs previously described [28] for editing the *Dmd* exon 10 locus was re-examined under different optimized experimental conditions using increased cell numbers and optimized program of Neon electroporation. The nine epegRNAs differed in their reverse transcriptase template (RTT) and primer binding site (PBS) lengths (Table 2) and were tested in combination with either PE-VQR or PE-SpG editors.

**Figure 3:**
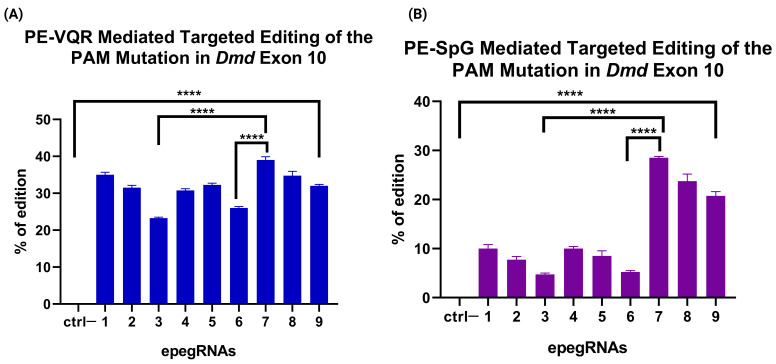
Optimization of epegRNA architecture for prime editing at the *Dmd* exon 10 locus in wild-type C2C12 myoblasts. (**A**) Editing efficiencies obtained using PE-VQR and nine epegRNAs differing in RTT and PBS lengths. Editing frequencies ranged from approximately 24% to 39%, with epegRNA-7 (RTT16/PBS10) producing the highest editing efficiency. (**B**) Editing efficiencies obtained using PE-SpG and the same panel of epegRNAs. Editing frequencies ranged from approximately 5% to 29%, with epegRNA-7 again exhibiting the highest activity. Editing efficiencies were determined by Sanger sequencing and EditR analysis 72 h after electroporation. Data are presented as mean ± SEM (N = 4 independent experiments). Statistical significance was assessed by one-way ANOVA followed by Tukey’s multiple-comparison test (**** *p* < 0.0001).

Editing outcomes were quantified by Sanger sequencing followed by EditR analysis. Using PE-VQR, all nine epegRNAs produced substantial editing, with efficiencies ranging from approximately 24% to 39% (Figure 3A). The highest editing efficiency was achieved with epegRNA-7 (RTT16/PBS10), which introduced the silent PAM mutation in approximately 39% of alleles. Several additional epegRNAs yielded editing frequencies above 30%, indicating efficient targeting of the *Dmd* exon 10 locus.

Editing efficiencies obtained with PE-SpG were generally lower than those observed with PE-VQR (Figure 3B). Editing frequencies ranged from approximately 5% to 29%, with epegRNA-7 again exhibiting the highest activity (~29%). Statistical analysis revealed significant differences among epegRNA designs, with epegRNA-7 outperforming several lower-performing constructs.

Comparison of the nine epegRNA architectures demonstrated that editing efficiency was strongly influenced by RTT and PBS length. In both PE-VQR and PE-SpG experiments, epegRNAs containing the longest RTT (16 nt) combined with the shortest PBS (10 nt) consistently produced the highest editing efficiencies. In contrast, increasing PBS length generally reduced editing performance, particularly when combined with shorter RTT sequences. These findings indicate that optimization of RTT and PBS architecture is a critical determinant of prime editing efficiency at the *Dmd* exon 10 locus.

Importantly, the RTT16/PBS10 design consistently yielded the highest editing efficiencies with both prime editor variants and was therefore selected for subsequent correction of the mdx5cv mutation in the engineered mutant myoblast cell line.

### 2.3. Prime Editing Mediated Correction of the mdx5cv Mutation

**Figure 4:**
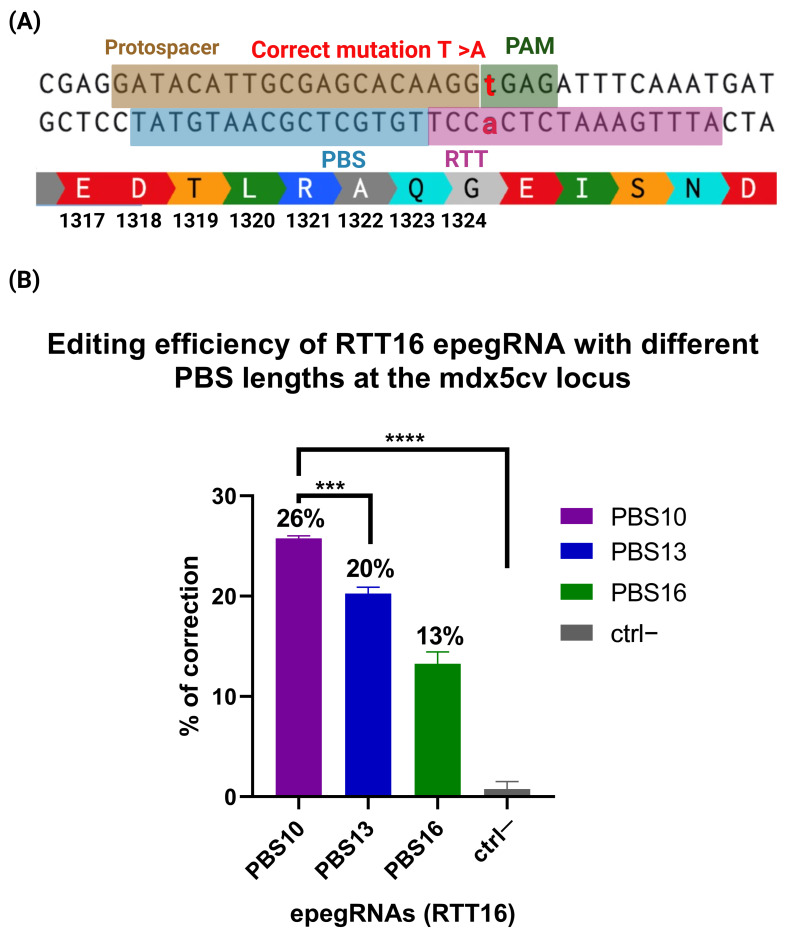
Optimization of epegRNA architecture for correction of the mdx5cv mutation. (**A**) Prime editing design for correction of the mdx5cv mutation in exon 10 of the mouse *Dmd* gene. The corrected nucleotide is indicated in red. (**B**) Correction efficiencies achieved with RTT16 epegRNAs containing PBS lengths of 10, 13, or 16 nucleotides. RTT16/PBS10 produced the highest correction efficiency, demonstrating the importance of PBS length in determining prime editing activity at the *Dmd* exon 10 locus. Data represent mean ± SEM (N = 4). Statistical significance was assessed by one-way ANOVA with Dunnett’s multiple-comparison test. *** *p* < 0.001, **** *p* < 0.0001.

Following the successful generation of a homozygous mdx5cv mutant C2C12 myoblast cell line, we next investigated whether prime editing could efficiently restore the wild-type *Dmd* sequence. Based on the optimization experiments described above, three epegRNAs sharing the optimal RTT length of 16 nucleotides but differing in primer binding site (PBS) length (10, 13, or 16 nucleotides) were selected for evaluation (Table 3). Each epegRNA was designed to correct the mdx5cv mutation while simultaneously introducing a silent PAM-disrupting mutation to prevent re-editing and facilitate tracking of editing events. Mutant myoblasts were electroporated with PE-VQR and the corresponding epegRNA constructs, and editing outcomes were quantified by Sanger sequencing followed by EditR analysis. No detectable editing was observed in the negative control (ctrl−), confirming the absence of background correction. In contrast, all three epegRNA designs successfully mediated correction of the mdx5cv mutation, with editing efficiencies ranging from approximately 13% to 26% (Figure 4). Among the tested designs, RTT16/PBS10 produced the highest correction efficiency, restoring the wild-type sequence in approximately 25–26% of alleles. Lower correction frequencies were obtained with RTT16/PBS13 (~20%) and RTT16/PBS16 (~13%), indicating that increasing PBS length negatively affected editing performance under these experimental conditions. 

Statistical analysis using one-way ANOVA revealed a highly significant difference among groups (*p* < 0.0001). Subsequent Dunnett’s multiple-comparison test demonstrated that all epegRNA-treated groups exhibited significantly higher correction frequencies than the negative control (**** *p* < 0.0001). Furthermore, RTT16/PBS10 consistently outperformed the other two epegRNA designs, highlighting the importance of epegRNA architecture in determining prime editing efficiency.

These findings demonstrate efficient and reproducible correction of the mdx5cv mutation in the engineered mutant myoblast cell line. Consistent with the PAM-modification experiments performed in wild-type C2C12 cells, the RTT16/PBS10 configuration remained the most effective epegRNA design, further confirming that a longer RTT combined with a shorter PBS provides optimal editing activity at the *Dmd* exon 10 locus. Collectively, these results establish RTT16/PBS10 as the lead candidate for subsequent differentiation of corrected mdx5cv C2C12 myoblasts into myotubes to evaluate dystrophin protein restoration.

### 2.4. In Silico Off-Target Analysis of Prime Editing Strategies Used for Generation and Correction of the mdx5cv Mutation

To evaluate the predicted specificity of the prime editing strategies used for both generation and correction of the mdx5cv mutation, potential off-target sites associated with the corresponding spacer sequences were identified using Cas-OFFinder [29,30] (http://www.rgenome.net/cas-offinder/) accessed from 4 March 2024 to 30 June 2026, against the mouse reference genome Mus musculus (mm10). The analysis was performed allowing up to three mismatches and excluding DNA or RNA bulges.

**Figure 5:**
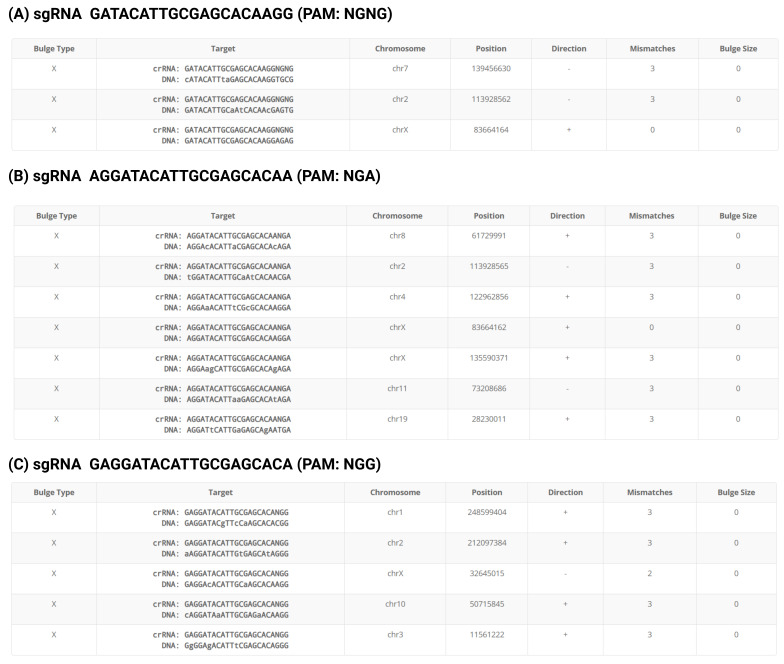
In silico off-target prediction of spacer sequences used for generation and correction of the mdx5cv mutation. (**A**) Predicted off-target sites for sgRNA GATACATTGCGAGCACAAGG (PAM: NGNG). (**B**) Predicted off-target sites for sgRNA AGGATACATTGCGAGCACAA (PAM: NGA). (**C**) Predicted off-target sites for sgRNA GAGGATACATTGCGAGCACA (PAM: NGG). Potential off-target sites were identified using Cas-OFFinder against the mouse genome (mm10), allowing up to three mismatches without DNA or RNA bulges. No candidate loci containing fewer than three mis-matches were identified for any of the three spacer sequences.

Potential off-target sites associated with the spacer sequences used for mutation generation and correction were predicted using Cas-OFFinder. No candidate off-target loci containing fewer than three mismatches were identified for either spacer sequence. All predicted off-target sites contained at least three mismatches relative to their corresponding on-target sequences (Figure 5), suggesting a favorable predicted specificity profile for both prime editing strategies. Although Cas-OFFinder analysis provides useful preliminary assessment of off-target risk, experimental validation using targeted deep sequencing or unbiased genome-wide off-target detection methods will be required to fully evaluate editing fidelity [31,32,33]. Nevertheless, the absence of closely matched genomic targets supports the suitability of these prime editing strategies for precise modification of the *Dmd* locus.

### 2.5. Differentiation of Corrected mdx5cv Myoblasts and Detection of Dystrophin Expression

**Figure 6:**
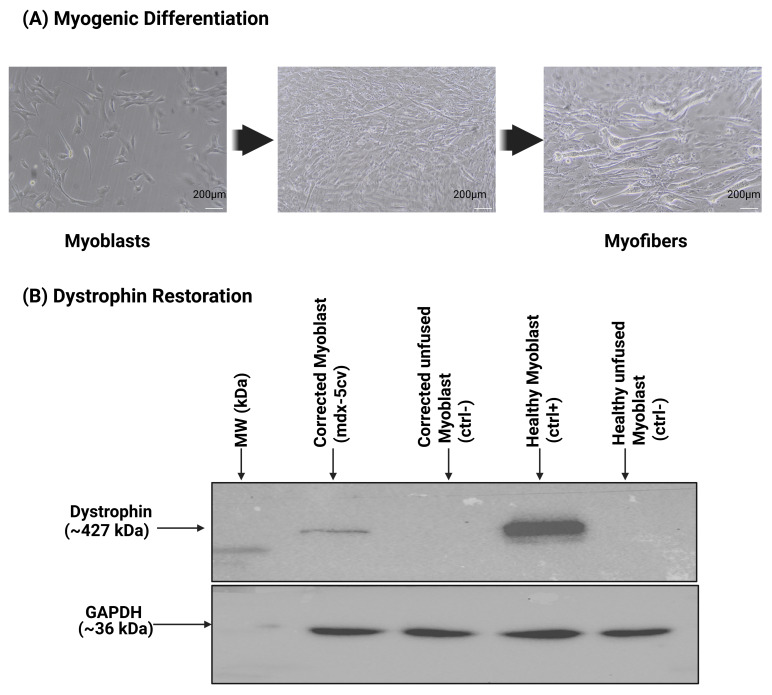
Differentiation of corrected mdx5cv myoblasts and restoration of dystrophin protein expression. (**A**) Representative bright-field images of corrected mdx5cv myoblasts following differentiation. Edited cells generated using the RTT16/PBS10 epegRNA fused efficiently to form elongated multinucleated myotubes, demonstrating preservation of myogenic differentiation capacity after prime editing. (**B**) Representative Western blot analysis of dystrophin expression. Protein extracts were prepared from differentiated corrected mdx5cv cells, and undifferentiated corrected mdx5cv cells, differentiated wild-type C2C12 myotubes, undifferentiated wild-type C2C12 myoblasts. A dystrophin band was observed in corrected mdx5cv myotubes and differentiated wild-type C2C12 myotubes, whereas no detectable dystrophin signal was observed in undifferentiated cells. Equal amounts of total protein (50 μg) were loaded for each sample. Following protein transfer, the membrane was cut horizontally according to molecular weight, with the upper portion probed for dystrophin and the lower portion probed for GAPDH, which served as the loading control.

To determine whether correction of the mdx5cv mutation was associated with restoration of dystrophin protein expression, the edited myoblast population generated using the most efficient epegRNA design (RTT16/PBS10) was expanded and induced to undergo myogenic differentiation. Following culture in differentiation medium, the corrected myoblasts efficiently fused to form elongated, multinucleated myotubes, demonstrating preservation of their myogenic potential after prime editing (Figure 6A). Microscopic examination confirmed the formation of mature multinucleated myotubes comparable to those observed in differentiated wild-type C2C12 cultures. To evaluate dystrophin expression, total protein extracts were prepared from differentiated and undifferentiated cell populations and analyzed by Western blot. Protein samples included differentiated wild-type C2C12 myotubes, undifferentiated wild-type C2C12 myoblasts, differentiated corrected mdx5cv cells, and undifferentiated corrected mdx5cv cells. GAPDH served as the loading control.

As expected, a dystrophin band was detected in differentiated wild-type C2C12 myotubes, whereas no detectable dystrophin signal was observed in undifferentiated wild-type myoblasts (Figure 6B). A dystrophin band was also detected in differentiated corrected mdx5cv cells following prime editing mediated correction of the mutation, whereas no detectable dystrophin signal was observed in undifferentiated corrected mdx5cv myoblasts. These representative Western blot results are consistent with the differentiation dependent expression pattern of dystrophin and indicate that correction of the mdx5cv mutation was associated with detectable dystrophin expression following myogenic differentiation. Collectively, these findings indicate that prime editing mediated correction of the mdx5cv mutation enabled dystrophin protein expression in differentiated myotubes. The detection of a dystrophin band in corrected differentiated cells provides biological support for successful correction of the pathogenic mutation and further identifies the RTT16/PBS10 epegRNA as the most effective design evaluated in this study.

## 3. Discussion

In the present study, we established a comprehensive prime editing workflow for both the generation and correction of the mdx5cv mutation in the mouse *Dmd* gene. Using PAM-flexible prime editor variants and optimized engineered pegRNAs, we first generated a homozygous mdx5cv myoblast cell line and subsequently corrected the pathogenic mutation, resulting in detectable dystrophin protein expression following myogenic differentiation. Together, these findings demonstrate the versatility of prime editing as a platform for both disease modeling and precise therapeutic genome correction in DMD [19,32].

A notable outcome of this work was the successful generation of a stable homozygous mdx5cv myoblast model through repeated prime editing and clonal isolation. While mdx mouse models remain indispensable for studying disease pathophysiology and evaluating therapeutic interventions, genetically defined cellular models provide complementary advantages for mechanistic and optimization studies. Such models enable controlled evaluation of guide RNA architecture, prime editor variants, delivery strategies, and functional rescue assays without the complexity and cost associated with animal studies. The ability to generate homozygous mdx5cv clones through prime editing without introducing double-strand DNA breaks further highlights the utility of this technology for precise engineering of disease-relevant cellular models.

A central finding of the present study was the strong influence of epegRNA architecture on editing efficiency. Optimization experiments demonstrated that editing outcomes at the *Dmd* exon 10 locus were highly dependent on the combination of reverse transcription template (RTT) and primer-binding site (PBS) lengths. Among the designs tested, the RTT16/PBS10 configuration consistently produced the highest editing efficiencies in both the PAM-editing and mutation-correction experiments. These findings are consistent with previous reports showing that prime editing efficiency is highly locus-dependent and that empirical optimization of pegRNA architecture remains essential for maximizing editing outcomes [20,34]. The superior performance of a longer RTT combined with a shorter PBS may reflect more favorable hybridization kinetics and reverse-transcription efficiency at this genomic locus. In addition, incorporation of a silent PAM-disrupting mutation likely contributed to improved editing outcomes by preventing repeated target recognition after successful editing while simultaneously providing a convenient molecular marker for tracking corrected alleles.

The correction of the mdx5cv mutation represents the principal therapeutic proof-of-concept of this study. Using the optimized RTT16/PBS10 epegRNA in combination with PE-VQR, correction frequencies of approximately 25–26% were achieved in the homozygous mutant myoblast line. Although editing efficiencies vary substantially across genomic loci and experimental systems, this level of correction is encouraging for a plasmid-based prime editing approach delivered by electroporation. Importantly, correction was achieved in a bulk cell population without enrichment or clonal selection of edited cells prior to functional analysis, suggesting that the observed dystrophin restoration reflects genuine editing activity within a heterogeneous population. These results further support the utility of the engineered mdx5cv cell line as a robust platform for evaluating future improvements in prime editor design, epegRNA engineering, and delivery technologies [25].

Beyond genomic correction, the detection of dystrophin protein in differentiated corrected mdx5cv myotubes provides important biological support for the editing outcome. Corrected mdx5cv myoblasts retained their capacity to differentiate and fuse into multinucleated myotubes, indicating that neither the prime editing procedure nor subsequent clonal expansion impaired their myogenic potential. Representative Western blot analysis showed detectable dystrophin expression in differentiated corrected cells, whereas no detectable dystrophin signal was observed in undifferentiated cells, consistent with the normal differentiation dependent expression pattern of dystrophin. Although the Western blot analysis was qualitative, these findings suggest that prime editing mediated correction of the pathogenic nucleotide substitution restored the ability of corrected cells to produce full length dystrophin following myogenic differentiation, thereby providing biological support for the genomic correction achieved.

The present work also contributes to the growing body of evidence supporting prime editing as a promising therapeutic strategy for DMD. Unlike conventional CRISPR/Cas9 approaches, prime editing enables precise nucleotide correction without donor DNA templates and without intentionally generating double strand DNA breaks. This distinction is particularly relevant for DMD, where unintended indels, large deletions, chromosomal rearrangements, and activation of DNA damage pathways may complicate therapeutic development [17]. Prime editing may therefore be especially attractive for the correction of point mutations, splice site mutations, and other small pathogenic sequence variants in which precise restoration of the endogenous gene sequence is desirable. Furthermore, the successful use of PAM flexible prime editor variants in this study highlights the importance of expanding target accessibility, as many clinically relevant mutations are not optimally positioned relative to canonical NGG PAM sites.

Genome editing specificity is a critical consideration for therapeutic development. To obtain a preliminary assessment of editing fidelity, potential off-target sites associated with the spacer sequences used for both generation and correction of the mdx5cv mutation were evaluated using Cas-OFFinder [29]. No candidate loci containing fewer than three mismatches were identified, suggesting a favorable predicted specificity profile for both editing strategies. Although computational prediction alone cannot exclude off-target activity, these findings support the suitability of the selected spacer sequences and provide a rationale for future validation using targeted or genome-wide sequencing approaches.

Several limitations of the present study should be acknowledged. Editing efficiencies were quantified using Sanger sequencing coupled with EditR analysis, which provides a rapid and cost effective method for initial optimization but lacks the sensitivity and resolution of next generation sequencing approaches [35]. Future studies should therefore employ deep sequencing to accurately quantify correction frequencies, bystander edits, indels, and allelic editing outcomes. Second, the experiments were performed in immortalized C2C12 myoblasts, and editing efficiencies may differ in primary myoblasts, satellite cells, skeletal muscle fibers, or cardiomyocytes. Although representative Western blot analysis demonstrated detectable dystrophin expression following prime editing mediated correction, the analysis was qualitative and did not include densitometric quantification. In addition, differentiated and undifferentiated uncorrected mdx5cv C2C12 cells were not included as negative controls in the Western blot analysis, which would have further strengthened the direct attribution of dystrophin restoration to prime editing mediated correction. Third, the long term stability of the corrected genotype and sustained dystrophin expression during extended cell culture and serial passaging were not evaluated. Finally, although in silico off-target prediction suggested a favorable specificity profile, comprehensive experimental validation will be required before clinical translation.

Future studies should focus on improving both editing efficiency and delivery. The engineered mdx5cv cell line developed here provides an excellent platform for evaluating next-generation prime editor variants, optimized epegRNA scaffolds, PE3 and PE5 based editing strategies, and alternative delivery modalities. In particular, non-viral delivery systems such as lipid nanoparticles (LNPs) [36], extracellular vesicles (EVs) [37] represent an attractive avenue for therapeutic development because they may facilitate transient expression of editing components while avoiding some of the limitations associated with viral vectors. Ultimately, validation of the optimized RTT16/PBS10 design in primary myogenic cells and in vivo DMD models will be required to determine its translational potential.

## 4. Materials and Methods

### 4.1. Cell Lines

The mouse myoblast cell line C2C12 was obtained from the American Type Culture Collection (ATCC, Manassas, VA, USA). This cell line was originally derived from the skeletal muscle of a C3H mouse following a crush injury and is widely used as an in vitro model to study myogenesis, muscle regeneration, and neuromuscular disease mechanisms. C2C12 cells retain a stable diploid genome and are amenable to genetic manipulation, making them suitable for evaluating genome-editing strategies targeting muscle-associated genes such as *Dmd*. All experiments were performed using low-passage cells to minimize genetic drift and phenotypic variability.

### 4.2. Plasmids

Prime editing plasmids pCMV-PE2 (Addgene #132775), pCMV-PE2-VQR (Addgene #159982), and pCMV-PE2-SpG (Addgene #159978) were obtained from Addgene, Watertown, MA, USA (https://www.addgene.org/). These plasmids encode a fusion protein consisting of an engineered SpCas9 H840A nickase and an M-MLV reverse transcriptase, enabling precise genome editing without generating double-strand DNA breaks.

The pU6-tevopreq1-GG-acceptor plasmid (Addgene #174038), which allows U6 promoter-driven expression of engineered prime editing guide RNAs (epegRNAs), was also obtained from Addgene. In our laboratory, this plasmid was further modified to include an additional U6 promoter and a cloning site for expression of a nicking single-guide RNA (nsgRNA) required for the PE3 strategy. This modified construct was designated pU6-epegRNA-nsgRNA.

Design of epegRNAs and nsgRNAs was performed according to the guidelines described by Anzalone et al. [19], with optimization of primer binding site (PBS) and reverse transcription template (RTT) lengths to maximize editing efficiency. Particular attention was given to avoiding premature transcriptional termination motifs within the epegRNA scaffold. All oligonucleotides used for cloning were synthesized by Integrated DNA Technologies (IDT, Coralville, IA, USA). Cloning was performed using standard restriction enzyme digestion and ligation or Golden Gate assembly, followed by sequence verification.

### 4.3. Cell Culture Conditions

Wild-type C2C12 myoblasts and genetically engineered mdx5cv C2C12 myoblasts were cultured in Dulbecco’s Modified Eagle Medium (DMEM; Wisent Inc., Saint-Jean-Baptiste, QC, Canada) supplemented with 10% fetal bovine serum (FBS) and 1% penicillin–streptomycin (both from Wisent Inc.) as previously described [28]. Cells were maintained at 37 °C in a humidified incubator with 5% CO_2_, and the culture medium was replaced every 2–3 days. Cells were passaged at approximately 70–80% confluence using standard trypsinization procedures.

For myogenic differentiation, confluent myoblast cultures were switched to differentiation medium consisting of DMEM supplemented with 1% FBS and 1% penicillin–streptomycin. The differentiation medium was replaced every 2–3 days until multinucleated myotubes were formed. All cell culture procedures were performed under sterile conditions.

### 4.4. Plasmid Electroporation in Mouse C2C12 Myoblasts

Plasmid delivery into C2C12 myoblasts was performed using the Neon™ Transfection System (Thermo Fisher Scientific, Waltham, MA, USA) as previously described [28], with modifications specific to the present study. Briefly, 100,000 cells were resuspended in Neon resuspension buffer and electroporated with a total of 2 μg of plasmid DNA per reaction, consisting of 1 μg of a prime editor plasmid (PE2, PE-VQR, or PE-SpG) and 1 μg of the corresponding epegRNA-nsgRNA plasmid (for PE3 experiments).

Electroporation was carried out using 10 μL Neon tips with the following parameters: 1300 V, 20 ms pulse width, and 2 pulses. Immediately after electroporation, the cells were transferred into one well of a 24-well plate containing 500 μL of pre-warmed culture medium. After 24 h, the medium was replaced with 1 mL of fresh DMEM to remove cell debris and residual electroporation buffer. Cells were incubated for an additional 48 h to allow sufficient time for prime editing to occur.

### 4.5. Negative Control Constructs

An engineered eGFP-expressing plasmid was used as a negative control and to assess electroporation efficiency [28]. Transfection efficiency was determined by fluorescence microscopy based on the percentage of eGFP-positive cells, which consistently exceeded approximately 80% under the optimized electroporation conditions. No *Dmd* targeting epegRNA was included in this control, and Sanger sequencing confirmed the absence of detectable editing at the *Dmd* locus. To ensure experimental consistency, all control and experimental groups were electroporated with an equal total amount of plasmid DNA (2 μg per reaction).

### 4.6. Genomic DNA Extraction and PCR Amplification

Genomic DNA extraction and PCR amplification were performed essentially as previously described [28]. Genomic DNA was extracted directly from cultured C2C12 cells using the DirectPCR™ Lysis Reagent (Viagen Biotech Inc., Los Angeles, CA, USA). Cells were washed once with 500 μL phosphate-buffered saline, followed by addition of 100 μL lysis reagent supplemented with 1 μL proteinase K (20 mg/mL). Samples were incubated at 56 °C for 2 h to ensure complete lysis and protein digestion, followed by enzyme inactivation at 85 °C for 45 min. Lysates were centrifuged at 13,000 rpm for 5 min, and 1–2 μL of the supernatant was used as template DNA for PCR amplification of the target genomic regions. PCR was performed using Phusion™ High-Fidelity DNA Polymerase (Thermo Fisher Scientific Inc., Waltham, MA, USA) to minimize amplification errors. Cycling conditions consisted of an initial denaturation at 98 °C for 30 s, followed by 35 cycles of 98 °C for 10 s, 62 °C for 20 s, and 72 °C for 30 s, with a final extension step at 72 °C for 30 s. Primer sequences were designed to flank the edited region and generate amplicons suitable for Sanger sequencing.

### 4.7. Sanger Sequencing and Editing Analysis

Sanger sequencing and editing analysis were performed essentially as previously described [28]. PCR products were submitted for Sanger sequencing to the CHU de Québec Research Center sequencing platform, Université Laval, Québec, Canada (https://sequences.ulaval.ca/murin/servseq.pageaccueil) accessed on 4 March 2024 to 30 June 2026. Sequencing reactions were performed using BigDye™ Terminator v3.1 chemistry (Thermo Fisher Scientific Inc.) with internal primers to ensure high-quality base calling across the edited site. Although amplicon deep sequencing provides higher sensitivity and quantitative resolution, Sanger sequencing combined with EditR analysis is a validated and widely used approach for estimating editing frequencies in initial optimization studies [35,38]. Sequencing chromatograms were analyzed to quantify prime editing efficiencies using the EditR online tool (https://moriaritylab.shinyapps.io/editr_v10/) accessed from 4 March 2024 to 30 June 2026 which enables robust estimation of nucleotide substitution frequencies from mixed Sanger traces. Editing efficiency was calculated as the percentage of modified alleles relative to the total signal at the target nucleotide position.

### 4.8. Western Blot Analysis

Differentiated C2C12 myotubes were lysed directly in the culture plates using 600 μL of lysis buffer. Total protein concentration was determined using a Qubit™ 4 Fluorometer (Invitrogen™, Thermo Fisher Scientific, Mississauga, ON, Canada) according to the manufacturer’s instructions. Equal amounts of total protein (50 μg per sample) were separated by 8% SDS-PAGE and transferred onto a PVDF membrane. Because of the large difference in the molecular weights of dystrophin (~427 kDa) and GAPDH (~36 kDa), the membrane was cut horizontally after transfer. The upper portion was incubated with a mouse monoclonal anti-dystrophin antibody (clone MANDYS8, Abnova, Taipei, Taiwan), whereas the lower portion was incubated with a mouse monoclonal anti-GAPDH antibody (clone 6C5, MAB374, Sigma-Aldrich/Millipore, Burlington, MA, USA). HRP-conjugated goat anti-mouse IgG (Thermo Fisher Scientific, Waltham, MA, USA) was used as the secondary antibody. Immunoreactive bands were detected using a chemiluminescent substrate and imaged with a ChemiDoc™ Imaging System (Bio-Rad Laboratories, Hercules, CA, USA). Representative Western blot images were used for the qualitative assessment of dystrophin expression in the different experimental groups.

### 4.9. Off-Target Prediction

Potential off-target sites for the spacer sequences used to generate and correct the mdx5cv mutation were predicted using the Cas-OFFinder [29,30] web tool (http://www.rgenome.net/cas-offinder/) accessed from 4 March 2024 to 30 June 2026 against the Mus musculus reference genome (mm10). The analysis was performed by allowing up to three nucleotide mismatches between the spacer and potential genomic target sites while excluding both DNA and RNA bulges. The spacer sequences corresponding to the prime editing guide RNAs used for mutation generation and correction were analyzed independently. Predicted off-target sites were ranked according to the number of mismatches and genomic location, and the resulting candidate loci were used to assess the predicted specificity of each prime editing strategy.

### 4.10. Statistical Analysis

All quantitative data were analyzed using GraphPad Prism version 8.0.2 (GraphPad Software Inc., La Jolla, CA, USA). Statistical significance was assessed by one-way ANOVA followed by Tukey’s multiple-comparison test. Asterisks indicate statistical significance (* *p* < 0.05, ** *p* < 0.01, *** *p* < 0.001, and **** *p* < 0.0001). Data are presented as mean ± standard error of the mean (SEM). N values represent independent biological replicates, defined as separate C2C12 cell cultures of the same cell line, each subjected to the full experimental workflow independently, including electroporation, culture, DNA extraction, and sequencing. Each experiment was performed under identical conditions to ensure reproducibility.

## 5. Conclusions

In summary, we established a prime editing-based workflow for both the generation and correction of the mdx5cv mutation in mouse C2C12 myoblasts. Repeated rounds of prime editing followed by clonal isolation enabled the generation of homozygous mdx5cv mutant cell lines, providing a genetically defined in vitro model for Duchenne muscular dystrophy. Optimization of epegRNA architecture identified the RTT16/PBS10 design as the most effective configuration for mutation correction, achieving approximately 26% restoration of the wild-type sequence. Importantly, corrected cells retained their myogenic differentiation capacity and restored dystrophin protein expression following differentiation into myotubes. In addition, in silico off-target analysis predicted a low risk of unintended genome modification, supporting the specificity of the selected editing strategies. Collectively, these findings demonstrate the utility of prime editing for both precise disease modeling and therapeutic gene correction and highlight its potential as a precision genome-editing approach for Duchenne muscular dystrophy. Future studies focusing on delivery optimization, comprehensive off-target validation, and in vivo evaluation will be essential for advancing this strategy toward clinical application.

## Figures and Tables

**Table 1:** Sequences and Characteristics of epegRNAs. The nucleotide A in red is to insert the mdx5cv mutation.

Names	Spacer Sequences	PBS Sequences	RTT Sequences	sgRNA Sequences for PE3
**(A) epegRNA optimization to create the mutated C2C12 myoblast (NGAG PAM)**				
epegRNA-1	AGGATACATTGCGAGCACAA	TGCTCGCAAT	TCTC**A**CCTTG	CAGTGTTTTCTTTTACCTCA
epegRNA-2	AGGATACATTGCGAGCACAA	TGCTCGCAATGTA	TCTC**A**CCTTG	CAGTGTTTTCTTTTACCTCA
epegRNA-3	AGGATACATTGCGAGCACAA	TGCTCGCAATGTATCC	TCTC**A**CCTTG	CAGTGTTTTCTTTTACCTCA
epegRNA-4	AGGATACATTGCGAGCACAA	TGCTCGCAAT	AAATCTC**A**CCTTG	CAGTGTTTTCTTTTACCTCA
epegRNA-5	AGGATACATTGCGAGCACAA	TGCTCGCAATGTA	AAATCTC**A**CCTTG	CAGTGTTTTCTTTTACCTCA
epegRNA-6	AGGATACATTGCGAGCACAA	TGCTCGCAATGTATCC	AAATCTC**A**CCTTG	CAGTGTTTTCTTTTACCTCA
epegRNA-7	AGGATACATTGCGAGCACAA	TGCTCGCAAT	TTGAAATCTC**A**CCTTG	CAGTGTTTTCTTTTACCTCA
epegRNA-8	AGGATACATTGCGAGCACAA	TGCTCGCAATGTA	TTGAAATCTC**A**CCTTG	CAGTGTTTTCTTTTACCTCA
epegRNA-9	AGGATACATTGCGAGCACAA	TGCTCGCAATGTATCC	TTGAAATCTC**A**CCTTG	CAGTGTTTTCTTTTACCTCA
**(B) epegRNA optimization to create the mutated C2C12 myoblast (NGAG PAM)**				
epegRNA-1	GATACATTGCGAGCACAAGG	TGTGCTCGCA	ATTTGAAATCTC**A**CCT	CAGTGTTTTCTTTTACCTCA
epegRNA-2	GATACATTGCGAGCACAAGG	TGTGCTCGCAATG	ATTTGAAATCTC**A**CCT	CAGTGTTTTCTTTTACCTCA
epegRNA-3	GATACATTGCGAGCACAAGG	TGTGCTCGCAATGTAT	ATTTGAAATCTC**A**CCT	CAGTGTTTTCTTTTACCTCA
**(C) epegRNA optimization to create the mutated C2C12 myoblast (NGG PAM)**				
epegRNA-1	GAGGATACATTGCGAGCACA	GCTCGCAATG	CTC**A**CCTTGT	GTACTTCTTCTAAAGCAGTT
epegRNA-2	GAGGATACATTGCGAGCACA	GCTCGCAATGTAT	CTC**A**CCTTGT	GTACTTCTTCTAAAGCAGTT
epegRNA-3	GAGGATACATTGCGAGCACA	GCTCGCAATGTATCCT	CTC**A**CCTTGT	GTACTTCTTCTAAAGCAGTT
epegRNA-4	GAGGATACATTGCGAGCACA	GCTCGCAATG	AATCTC**A**CCTTGT	GTACTTCTTCTAAAGCAGTT
epegRNA-5	GAGGATACATTGCGAGCACA	GCTCGCAATGTAT	AATCTC**A**CCTTGT	GTACTTCTTCTAAAGCAGTT
epegRNA-6	GAGGATACATTGCGAGCACA	GCTCGCAATGTATCCT	AATCTC**A**CCTTGT	GTACTTCTTCTAAAGCAGTT
epegRNA-7	GAGGATACATTGCGAGCACA	GCTCGCAATG	TGAAATCTC**A**CCTTGT	GTACTTCTTCTAAAGCAGTT
epegRNA-8	GAGGATACATTGCGAGCACA	GCTCGCAATGTAT	TGAAATCTC**A**CCTTGT	GTACTTCTTCTAAAGCAGTT
epegRNA-9	GAGGATACATTGCGAGCACA	GCTCGCAATGTATCCT	TGAAATCTC**A**CCTTGT	GTACTTCTTCTAAAGCAGTT

**Table 2:** Sequences and characteristics of the nine epegRNAs used for editing the *Dmd* exon 10 locus. epegRNAs differed in reverse transcription template (RTT) and primer-binding site (PBS) lengths**.** The nucleotide G in red is to insert a traceable silent mutation, and the T in green is to correct the mdx5cv mutation.

epegRNAs	RTT Length (nt)	PBS Length (nt)	RTT Sequences	PBS Sequences
1	10	10	AATCT**GT**CCT	TGTGCTCGCA
2	10	13	AATCT**GT**CCT	TGTGCTCGCAATG
3	10	16	AATCT**GT**CCT	TGTGCTCGCAATGTAT
4	13	10	TGAAATCT**GT**CCT	TGTGCTCGCA
5	13	13	TGAAATCT**GT**CCT	TGTGCTCGCAATG
6	13	16	TGAAATCT**GT**CCT	TGTGCTCGCAATGTAT
7	16	10	ATTTGAAATCT**GT**CCT	TGTGCTCGCA
8	16	13	ATTTGAAATCT**GT**CCT	TGTGCTCGCAATG
9	16	16	ATTTGAAATCT**GT**CCT	TGTGCTCGCAATGTAT

**Table 3:** Characteristics of the epegRNAs evaluated for correction of the mdx5cv mutation in homozygous mutant C2C12 myoblasts. All epegRNAs contained an identical RTT length (16 nt) and differed only in PBS length. The nucleotide T in green is to correct the mdx5cv mutation.

epegRNAs	RTT Length (nt)	PBS Length (nt)	RTT Sequences	PBS Sequence
1	16	10	ATTTGAAATCTG**T**CCT	TGTGCTCGCA
2	16	13	ATTTGAAATCTG**T**CCT	TGTGCTCGCAATG
3	16	16	ATTTGAAATCTG**T**CCT	TGTGCTCGCAATGTAT

## Data Availability

The original contributions presented in this study are included in the article. Further inquiries can be directed to the corresponding author.
